# The Taxonomy of Blockchain-based Technology in the Financial Industry

**DOI:** 10.12688/f1000research.133518.1

**Published:** 2023-05-02

**Authors:** Andry Alamsyah, Syahputra Syahrir

**Affiliations:** 1 School of Economics and Business, Telkom University, Bandung, West Java, Indonesia

**Keywords:** Blockchain, Financial Innovation, Decentralization, FinTech, Decentralized Finance, Review

## Abstract

The decentralized approach of blockchain technology has resulted in innovations across various industries, including finance which is facing challenges due to the rise of decentralized finance (DeFi) in the market. Decentralization improves business processes and spurs product innovation through increased transparency and removing intermediaries. A taxonomy, created through literature review and expert interviews, outlines the four dimensions of these advancements: key drivers, products, benefits, and emerging threats. Proposed solutions are also included to tackle the threats.

## 1. Introduction

The financial sector is one of the largest in the world, and as of Q1 2022, the market cap of the top 100 banks globally was 6.1 trillion USD. ^1^ Technological advancements have had a major impact on the financial industry, leading to the rise of Financial Technology (FinTech) companies. FinTech refers to financial companies that utilize technology, mainly internet-related as the media, to launch products/services and to innovate. ^2^ This technology can also bring new products to market with improved security, efficiency, flexibility, and opportunities. An example is using technology to make credit card payments with smartphones and tablets, ^3^ increasing payment efficiency by allowing access to the user’s device without carrying the card or cash.

FinTech has a long history in the financial industry development. The evolution of FinTech consists of three digitalization phases, shown in Table 1 ^2^ and explained in the subsequent paragraph.

**Table 1.** Evolution Step of FinTech.

Phase	Name	Timeline	Strategy focus	Notable products/services
1	Internal	1960-2010	Internal process	Automatic Teller Machine (ATM)
2	Provider-oriented	2011-2020	Provider's integration	International Organizations for Standardization (ISO)
3	Customer-oriented	2021-now	Customer's service	Peer-to-peer business model

The first digitalization phase, called “internal,” concentrates on enhancing financial institutions’ internal operations by automating financial products and services. An example of this phase is the ATM, which allows customers to access banking services without visiting the bank. The second phase of digitalization is called provider oriented. It focuses on the provider’s integration by standardizing the processes and applications across different functions in the company. The goal is to establish a synchronized business process, resulting in a lower in-house production degree. The last phase is known as customer-oriented digitalization. As the name suggests, it emphasizes the services around the customer needs, which would establish a new financial ecosystem. Users are able to conduct peer-to-peer transactions without the third party’s involvement.

Blockchain is a technology capable of disrupting business processes and triggering innovations. But first, we need to know what blockchain is. It refers to a ledger secured by a chain of blocks guarded by cryptograpic techniques to secure its integrity. ^4^ Blockchain implementation covers two sectors, the financial and the non-financial industry. ^5^ The revolution brought by blockchain to the organizational structure is through Decentralized Autonomous organization (DAO). DAO offers a decentralized system rather than a pyramid hierarchy, which is often used in conventional institutions. The decentralization also drives the innovation of numerous new products, eliminating intermediaries’ functions. For instance, blockchain is the technology behind the worldly-renowned Bitcoin, which Satoshi Nakamoto proposed back in 2008. ^6^ Blockchain is a staggering technology with a decentralized mechanism that enables peer-to-peer payment. In other words, it could unlock payment without the interference of a third-party. The blockchain utilizes two key drivers: The Smart Contract and a Distributed Ledger Technology (DLT). Smart Contracts have unalterable terms, so both parties must carefully review them before signing. DLT records transactions more securely than centralized systems by sharing records among multiple nodes and using a consensus algorithm to validate data. The DLT ensures all nodes have the latest updated information.

The decentralization idea of blockchain drives new product innovation, allowing stakeholders to interact directly without the interference of a central authority. In finance, we can imagine if financial activities, such as saving, lending, and lending, are made between individuals. The decentralization idea drives the establishment of Decentralized Finance (DeFi). DeFi enables its users to manage their virtual assets, unlike the conventional financial institution that manages the user’s assets. Another blockchain product in the financial sector is Centralized Finance (CeFi). However, it doesn’t inherit the essence of blockchain’s decentralized trait since CeFi is an institution that manages its users’ assets. Therefore, we will focus on the discussion of DeFi and its byproducts. Our study aims to provide a classification of blockchain’s integration in finance. The first section covers the introduction, which discusses the financial industry and blockchain background. The second section concerns the literature review that formed the methodology of this review article. The third section presents the taxonomy based on the dimensions of key drivers, benefits, products, and threats. The fourth section addresses potential solutions to threats. The two final sections discuss and conclude the paper. Finally, we hope the taxonomy enlightens academicians and financial industry practitioners about the blockchain technology ecosystem.

## 2. Literature review

This paper employs an extensive literature review as its research methodology. The literature review thoroughly examines numerous scholarly works on blockchain technology and its practical applications. For instance, the investigation conducted by Zhang and Chan ^7^ inspection energy consumption in blockchain consensus algorithms, while Tarr’s research ^8^ delves into blockchain applications within the insurance sector. Blockchain technology is applicable across a multitude of industries. However, despite its vast potential for implementation, a limited number of enterprises have adopted it, primarily due to its nascent state. The literature review examines 73 academic articles from various reputable sources, including Elsevier, Emerald, MDPI, IEEE, ACM, and Taylor & Francis. The pertinent keywords encompassed blockchain technology, application, implementation, and terms related to blockchain products in the financial industry. Additionally, we have collected information from various crypto projects, exchanges, crypto industries, wallet services, crypto platforms information, and regulators/central bank through their website, whitepapers, and source code.

Based on the member’s participation, the blockchain is divided into permissioned and permissionless blockchains. ^9^ The permissioned blockchain only allows a particular authorized party to access information and participate in the system. In contrast, everyone can enter and oversee the information contained in the permissionless blockchain system. Financial institutions can choose whether to use one or both blockchain types based on their needs. For instance, to secure privacy, an insurance company implements the permissioned blockchain to prevent data exposure to the public. Blockchain has two key drivers: Smart Contracts and DLT. The Smart Contract refers to a legal contract capable of being implemented and expressed in the software. ^10^ In other words, a directly coded self-executing terms of agreements between buyer and seller. ^11^ The Smart Contract works by inserting code into the blockchain; as we know, the code embedded in the blockchain is unaltered (immutable) once deployed to the network and can only operate once the terms are met. The second key driver of blockchain is DLT. DLT contains multiple copies of transactions distributed among numerous participants and updated by the parties’ consensus. ^12^ This technology synchronizes information across multiple nodes, ensuring each node has the most recent ledger update.

Blockchain technology’s decentralization distinguishes it from conventional systems. Centralized systems store records on a single server with backups on additional devices, while decentralized systems employ consensus algorithms to synchronize records across multiple devices. Notable consensus algorithms include Proof of Work (PoW) and Proof of Stake (PoS). PoW rewards miners based on their capacity to solve network-generated mathematical problems, with greater computational power increases the likelihood of reward. Bitcoin, the preeminent cryptocurrency, utilizes PoW, which aims to prevent any miner from possessing over 50% of total computational power to maintain network integrity. Conversely, PoS rewards miners according to the quantity and duration of coins held within the network, ^13^ making it difficult for malicious actors to gain control, as doing so requires significant investment. PoS is considered more secure and energy-efficient than PoW, as it selects network leaders based on the stake and time commitment, reducing energy consumption compared to PoW’s reliance on substantial computational power. ^14^

Blockchain technology’s attributes can revolutionize FinTech firms’ operations, encompassing security and governance. “Security” pertains to properties such as immutability, transparency, traceability, data record, and tokenization. For example, FinTech firms can facilitate enhanced procurement agreements via Smart Contracts. The second attribute involves modifying companies’ governance systems and decentralizing organizational structures to optimize business processes. Voluntariness, equality, and mutual benefit characterize relationships between nodes in the blockchain’s governance system.

Blockchain tokenization allows users to manage digital assets more decentralized than traditional methods. Tokenization entails digitalizing assets to facilitate fractional investment and ownership. ^15^ These tokens can be traded or transferred like conventional securities. ^11^ Tokenization-driven economies replace intermediaries with technical protocols, ^16^ streamlining asset tracking for origin, authenticity, and rights. ^17^ Tokenized assets extend beyond physical items to intangible assets such as Non-Fungible Tokens (NFTs). For example, someone owning 30% of a digitalized yellow dog NFT, has the right to sell his NFT, limited to his 30% ownership stake.

DeFi embodies the core principles of blockchain in the FinTech sector. This paper focuses on DeFi’s discussion since it portrays the essence of blockchain: decentralization. However, Centralized Finance (CeFi) is also classified as a blockchain-based FinTech byproduct. DeFi lets users take care of their financial assets. Meanwhile, CeFi refers to a financial institution that controls its users’ financial assets. ^18^ DeFi combines one or more elements: decentralization, DLT and blockchain, smart contracts, disintermediation, and open banking. ^19^ On the other hand, examples of CeFi are companies like *Binance, Coinbase,* and *Uphold.*

Despite the various advantages blockchain technology offers, it is not flawless. Blockchain could cause new threats that come from both internal and external factors. Those threats might eventually be harmful to financial institutions if not appropriately handled. However, researchers have made efforts to address these risks, which we will outline in section 4. We have identified five specific threats to the implementation of blockchain in the financial industry, which include: scalability and speed, security, cost, regulation, environment, and energy.

Scalability and speed present significant challenges for blockchain implementation. In contrast to Visa’s capacity for around 2000 transactions per second, Bitcoin’s blockchain processes a mere seven transactions per second. ^20^ The generation of only 1 MB of data every 10 minutes ^21^ limits transaction volume. Storage and network capacity further hinder scalability and speed, as data generated by multiple devices must be processed and stored on the network, leading to block propagation delays and high resource consumption. Despite FinTech benefits, blockchain remains susceptible to security threats. The PoW algorithm enables the 51% attack, where malicious parties control over 50% of the total computational power, potentially dominating the blockchain and implementing rules to their advantage. ^22^ Phishing poses another security threat, ^23^ as fake wallets on popular apps allow scammers to seize victims’ funds by controlling private keys.

Even though blockchain can reduce the intermediaries cost through decentralization, it causes a new problem. The limited size of a blockchain system causes the miner to put in a higher mining fee. Moreover, the larger the transaction being sent, the higher the fees. The miners of blockchain, especially Ethereum, are being paid in the form of gas instead of fiat currency. The Gas price refers to the fees required to perform the computational effort. ^24^ The calculation of the total transaction fee in Ethereum is the gas limit multiplied by the gas price. ^25^ The gas limit refers to the maximum quantity of gas the creator is eager to spend, while the gas price determines the amount of Gwei the creator is willing to pay. ^26^ For the record, Wei is the smallest part of Ether, where 1 Ether equals $1^{0.18}$ Wei, whereas 1 GWei equals 1,000,000,000 Wei. ^25^ Over time and with the surging popularity and high demand for blockchain, the gas cost keeps increasing to the point where Ethereum has to manage millions of transactions daily.

Weak regulations could increase the risk of criminality. For instance, criminals use blockchain-based assets such as cryptocurrency to launder money and cease the money’s origin. There are two reasons blockchain-based digital assets are prone to the money laundering act. ^27^ The first is the governance of global anti-laundering agencies only worried about illegal financial flows and transactions and whether they meet standard theoretical money. Fiat money and blockchain-based digital assets are treated the same in tackling the money laundering act. The second reason is the novel manner digital asset offers, such as verification, undertaking, and transaction publication, are done almost in real-time.

Blockchain could consume more energy than traditional technology. For the record, the estimation to mine 1 Bitcoin requires the same energy to generate electricity for one US household for two years. ^28^ It indicates the enormous energy consumption of blockchain, consequently leaving more carbon footprints on the earth. A consensus algorithm such as PoW requires lots of power since the reward for the miner will be given when they find a new block in the network. ^29^ The increased work rate will consume energy which will eventually damage the environment. The blockchain’s energy consumption is often criticized due to its impact on the surroundings. As the title suggests, our paper’s discussion focuses on the impact of blockchain on the financial industry. We do not highlight blockchain’s implementation in other industries. However, we highlight how blockchain technology works in general. It is done to give an overview to the readers regarding the broad understanding of how blockchain works before pursed it to its implementation in the financial industry.

## 3. The taxonomy

**Figure 1.**
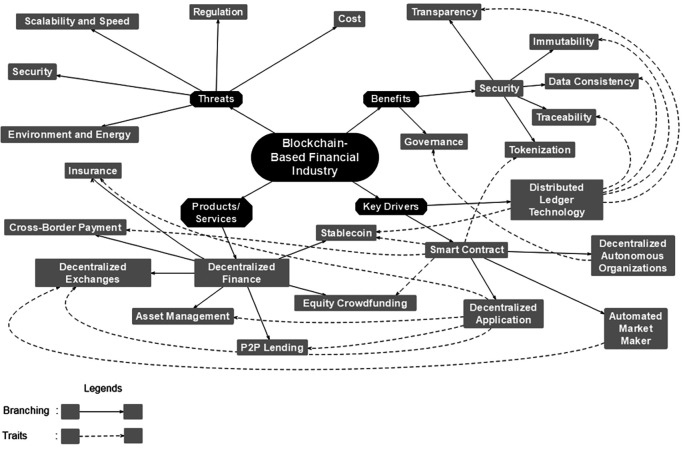
Taxonomy of blockchain-based financial industry.

Based on the discussion in the literature review section, our finding divides the taxonomy into four dimensions: key drivers, benefits, products/services, and threats ( Figure 1) represents the taxonomy:

We thoroughly discuss each of those four dimensions, from key drivers to threats. In addition, we analyze the dimension’s relationship to understand how one could affect another. The bold line represents the branching of its original dimensions. For instance, the Smart Contract consists of three branches: Decentralized Autonomous Organizations (DAO), Decentralized Applications (DApp), and Automated Market Makers (AMM). The dotted line represents the traits of the originator. For example, DLT traits are transparency, immutability, data consistency, and traceability.

### 3.1 Key drivers

Two important drivers are identified as the core of blockchain technology capabilities in supporting many complex interactions in the financial industry. They are Smart Contracts and DLT. Smart Contracts act as an unbiased and precise alternative to human decision-making, while DLT provides consistent records, transparency, and a robust database. Here is the discussion of each driver.

**3.1.1 Smart Contract**

A Smart Contract refers to a self-executing program that automatically executes and enforces the contract’s terms. Since it is written on the blockchain, it inherits its characteristics, such as being immutable or cannot be modified, tamper-proof, and transparent. Once a Smart Contract is created, it can be executed on the blockchain network and applied to any actor in the network. The Smart Contract can be substitute intermediaries in the financial industry, enabling decentralized transactions. ^31^ This means that users can carry out transactions with each other directly, rather than relying on a financial institution to set the terms of the trade. The peer-to-peer interaction characteristic in the financial industry is commonly known as DeFi. Furthermore, Smart Contracts create a financial ecosystem in which users have the power to manage their assets. While financial companies still exist in the DeFi space, they only serve as platform providers to facilitate asset trading among users and do not actually hold any assets themselves.

The Smart Contract enables unaltered and globally enforced agreements between parties since it has a synchronized record. The Smart Contract’s code is self-executing once it meets the agreed conditions. For instance, a company uses a Smart Contact to schedule the employee’s payroll. The only requirement is when it reaches the 25 ^th^ day of each month. Thus, the performance and other factors are not embedded in the contract. As a result, the employees will automatically receive their salary on the 25 ^th^ day of the month, despite their bad performance and other negative factors. A Smart Contract is the base principle for several technologies. These include DAO, DApp, and AMM, which we explain in the following subsection.

**3.1.1.1 Decentralized Autonomous Organization**

A decentralized Autonomous Organization, also known as DAO, is a blockchain-based system that allows entities in the organization to govern and coordinate themselves by a set of registered rules. ^30^ DAO can transform the organization’s hierarchical structure into a decentralized one. Conventional corporate governance contains two fundamental problems. ^31^ Everyone in the organization may not always be obedient to the rules, and others may not always agree with the rules. Fortunately, DAO possesses the ability to overcome those problems with the following characteristics ^32^:

1. The absence of hierarchical structure and central authority. It means the connections between nodes are not controlled by managerial association but rather by the value of voluntariness, equality, and mutual benefit.
2. The distributed organizations. Stakeholders arrange all the rules and collaboration patterns in DAO. Therefore, the DAO implementation would increase trust and reduce communication and transaction costs.
3. The reliance on the Smart Contract. The Smart Contract enhances DAO’s transparency and openness regarding participants’ responsibility, authority, operational rules, penalties, and rewards.

DAO allows the organization to work in synchronized continuously with the help of computer code or Smart Contract. DAO has become a fundamental concept of how governance does not necessarily need a central authority to function correctly, thus, it promotes transparency, democratic decision-making, and organizational efficiency. When large crowds participate in community or project initiatives, it can be more effective and engaging if decisions or actions are automatically arranged while maintaining order.

**3.1.1.2 Decentralized Application**

DApps represent practical implementations of DAOs. DApps distribute authority and control among users rather than a single institution. There are three primary application types: centralized, distributed, and decentralized. Centralized applications have a single controlling authority, distributed applications spread information across multiple nodes, and decentralized applications lack a single control point. DApps exhibit four main features ^33^:

1. A DApp is an open-source application, allowing for transparency within the network. However, it is vulnerable to plagiarism as the content is accessible to anyone in the network.
2. DApp developers engage in economic activities by creating a coin that they can control the distribution and supply of, and its value is determined by public perception.
3. To validate transactions within DApps, the implementation of blockchain and a consensus algorithm is essential. Transactions are recorded immutably in the blockchain, ensuring their history and preventing them from being altered by malicious parties.
4. DApps are designed to avoid the effect of server shutdown by ensuring that another node takes over the application’s functions if one node fails.

**3.1.1.3 Automated Market Maker**

AMM is an innovative approach to the traditional market that employs a third party to operate the order book. AMM is an algorithm or Smart Contract that enables importation and participation in an electronic market. ^34^ The main benefit of AMM utilization is liquidity, which refers to the ability to convert assets into cash without significantly impacting the market price. AMM works as a single-function algorithm that matches trading orders and determines execution prices. ^35^ Thanks to a liquidity pool, it provides superior liquidity than a conventional order book. A liquidity pool works as a vault, or a “pool”, where the liquidity provider (investor) puts one or many tokens for the trader so they can use it in many ways, such as lending, borrowing, derivatives, insurance, and swapping. ^36^ By conducting a transaction in the liquidity pool, the trader must pay the service fee, which is then transferred to the liquidity provider as the reward for providing the liquidity. The AMM covers numerous blockchain-based FinTech companies such as *Uniswap, Balancer, PancakeSwap, Osmosis,* and *1inch.*

**3.1.2 Distributed Ledger Technology**

The DLT records, stores, and shares information across the network of computers, thus making it more resilient compared to the centralized ledger. ^37^ The DLT’s characteristic brings nodes synchronized with the latest information update across the network. Therefore, if one node fails to receive updated information, another node will inform it. For example, a centralized system backs the data every hour and records it an hour later. The system records the transaction at 07.00 AM and backups data at 08.00 AM. However, a system malfunction occurred at 7.30 AM, forever changing every data from 07.00-07.30 AM. In contrast, the decentralized system synchronizes the transaction record simultaneously to every node in the system. DLT consists of three main layers. ^38^

1. Foundation. It refers to the entire DLT system’s foundation. It describes the rules on how the system governs the whole network in distributing the information.
2. Network. It’s a network where interconnected nodes or actors share, store, and process data.
3. Data. As the name suggests, the layer refers to the data stored in the DLT system as records.

### 3.2 Benefits

Innovative technology, like blockchain, is crucial for businesses to stay competitive in today’s ever-evolving market. Blockchain has the potential to revolutionize operations, streamline processes, increase efficiency, reduce costs, and improve overall performance. Its implementation in FinTech companies provides crucial benefits such as enhanced security and governance, making it a valuable addition to the taxonomy.

**3.2.1 Security**

The improved security in a financial system would result in a condition where the institutions and their users can conduct activities without worrying about their assets being stolen or information being compromised. The blockchain’s features, namely the Smart Contract and DLT, are crucial in improving FinTech’s security sector. The Smart Contract establishes the agreement between two or more parties and only becomes effective once the conditions agreements are met. DLT, on the other hand, synchronizes the record to each authorized node simultaneously. Generally, what makes blockchain brings a high degree of appeal when it comes to security while still maintaining privacy is the ability to support several properties such as immutability, transparency, traceability, data consistency, tokenization, and governance. In the financial industry scenario, A FinTech company could easily release fully digital products/services which can be guaranteed to perform similarly to the conventional product but with the flexibility of digital nature.

Another security aspect incorporated in blockchain features is Public Key Infrastructure (PKI). PKI basically uses public key cryptography to secure transactions and provide privacy and authentication. Each user has a public key and a private key. The public key is used to receive transactions, while the private key is used to sign transactions. When a user initiates a transaction, they sign it with their private key, and the transaction is broadcast to the network. Other nodes on the network can then verify the transaction by using the sender’s public key. A digital signature is used to provide authenticity and transaction integrity. The digital signature is generated by using the sender’s private key to encrypt the transaction data. The signature can then be verified by using the sender’s public key.

In summary, there are three layers of mechanisms to enhance security in the blockchain:

1. Hashing and encryption. The purpose of hashing is to connect every node to attain a synchronized record. The node is encrypted using a private or public key.
2. Consensus algorithm. It selects the blockchain’s participants, so the transaction validators are only the selected ones. The types of consensus algorithms may vary among different blockchain
3. Network consensus. It refers to the community’s decision to decide the latest consensus state. Therefore, any suspicious transaction would be ignored.

**3.2.1.1 Immutability**

Immutability refers to a condition where the transactions are unaltered once they are verified and recorded in the system. ^39^ The distributed nature of the record across the network poses significant challenges for any potential attacker looking to manipulate it, as they would need to replace each record with their own version of events. Thus, blockchain guarantees the single source of truth of a network. As the network expands in size, the possibility of an attacker replacing the records on every node becomes increasingly infeasible. As such, immutability provides a guarantee of information security, and is a fundamental characteristic that underpins the trustworthiness of the Smart Contract. Consequently, parties engaging with the Smart Contract must diligently read the contract terms before agreeing to them, as once launched to the system, no party can alter them. The Smart Contract’s immutability feature enhances the trust between parties, obviating the need for constant monitoring to ensure compliance with the agreed-upon terms.

**3.2.1.2 Transparency**

Transparent data would result in improved traceability, consistency, and immutability. Blockchain enables parties to share replicated ledgers, which act as a trusted record system and the sole source of truth of activities or transactions. ^40^ The blockchain’s DLT allows anyone or a particular party(es) to oversee the transaction activity. The network may determine who has the right to observe the data by choosing whether to use the permissioned or permissionless blockchain. Data transparency has become an essential key in establishing trust between stakeholders. In DeFi, where peer-to-peer transactions are encouraged, transparency is fundamental in establishing trust and stakeholder reputations. As no central authority can verify the stakeholder’s legitimacy, it is impossible to endorse an untrustworthy stakeholder in DeFi.

**3.2.1.3 Traceability**

Traceability refers to a system’s ability to trace and track the history of transactions, interactions, or movements. The demand for traceability information has recently increased among companies, consumers, and governments, as it is linked to the quality and safety of products and services. Therefore, utilizing blockchain technology provides a feasible solution to address these concerns. The blockchain’s DLT is the crucial reason the system’s information is easily trackable since the block contains information from the previous block. The recorded history plays a vital role for any stakeholder to have a full picture of any item, such as digital assets representing financial products/services. Therefore, traceability unmasks the flow of information. ^41^ It comes from the fact that DLT is a decentralized system where the information contained in the system is visible to all participants. ^42^ Every node in the DLT includes a complete record of the transactions, thus increasing the traceability of the asset’s movement.

**3.2.1.4 Data consistency**

Data consistency refers to the ability of blockchain technology to ensure that all copies of information on each stakeholder are identical and up-to-date. This requires record synchronization throughout the operation. It implies that all nodes in the network share the same information, ensuring the information’s consistency and accuracy throughout the network. The improved data consistency originated from DLT implementation, where the transactions are recorded and distributed simultaneously to every node. Hence every node has the latest transaction update. Moreover, participants in the system can notice the double-spending issue, ^43^ a flaw where money or assets are spent more than once.

**3.2.1.5 Tokenization**

Tokenization converts physical assets into digital tokens using blockchain technology, ensuring security, robustness, and privacy protection. As financial instruments and products are essentially abstractions, it makes sense to represent them as digital assets. Tokenized asset ownership is embedded within the system, allowing owners to view transaction records and identify buyers and sellers. Tokenized assets can be derived from tangible or intangible assets, such as artwork, precious stones, airplanes, intellectual property, or company equity. There are three primary benefits of tokenizing an asset ^44^:

1. Fractional ownership: Facilitates participation from retail and small investors.
2. Efficiency enhancement: Streamlines transactions, contracts, interest, and dividend execution, while minimizing intermediary involvement.
3. Improved transparency: Offers greater visibility into the asset’s history and transactions.

**3.2.2 Governance**

Governance refers to the rules, processes, and decision-making mechanisms that govern the operation and management of organizations, communities, and crowds. Blockchain technology advocates the decentralized mechanism throughout their operation. As the opposite of centralized and hierarchical operations, each stakeholder has their share proportions of the organization’s decision based on the agreed-upon preliminary rules or protocol. Therefore, societies and interactions are governed by various actors’ networks. ^43^ Blockchain-based governance aims to balance accountability and decentralization to ensure the organization’s long-term sustainability. Blockchain governance consists of on-chain and off-chain governance. ^45^ The on-chain governance explicitly defines the governance arrangement in the protocol, allowing stakeholders to vote or make changes to the proposal. Off-chain governance refers to the external governance structure protocol. DAO represents the implementation of decentralized corporate governance, which utilizes tokenized tradable shares to give network participants the weight of voice in governing the organization. It could function to provide dividends to shareholders. ^30^

### 3.3 Products/services

Recent technological advancements have led to the development of innovative products offering various features and benefits. In the financial industry, the core idea of enabling peer-to-peer transactions has led to the creation of decentralized finance, decentralized exchange, P2P lending, equity crowdfunding, stablecoins, and more. These products have been developed due to key drivers, features, and benefits offered by technology.

**3.3.1 Decentralized finance**

DeFi, a blockchain product in the financial industry valued at over fifty billion US dollars, challenges the fundamentals of traditional finance by removing intermediaries. ^19^ Smart Contracts replace these intermediaries, acting as transaction supervisors. Though financial institutions exist in the DeFi ecosystem, they primarily serve as platforms for user activities. Traditional financial institutions’ drawbacks, such as complex service policies, high costs, and limited transparency, are mitigated through DeFi, enabling peer-to-peer transactions, reduced bureaucracy, lower costs, and a transparent ecosystem.

**Figure 2.**
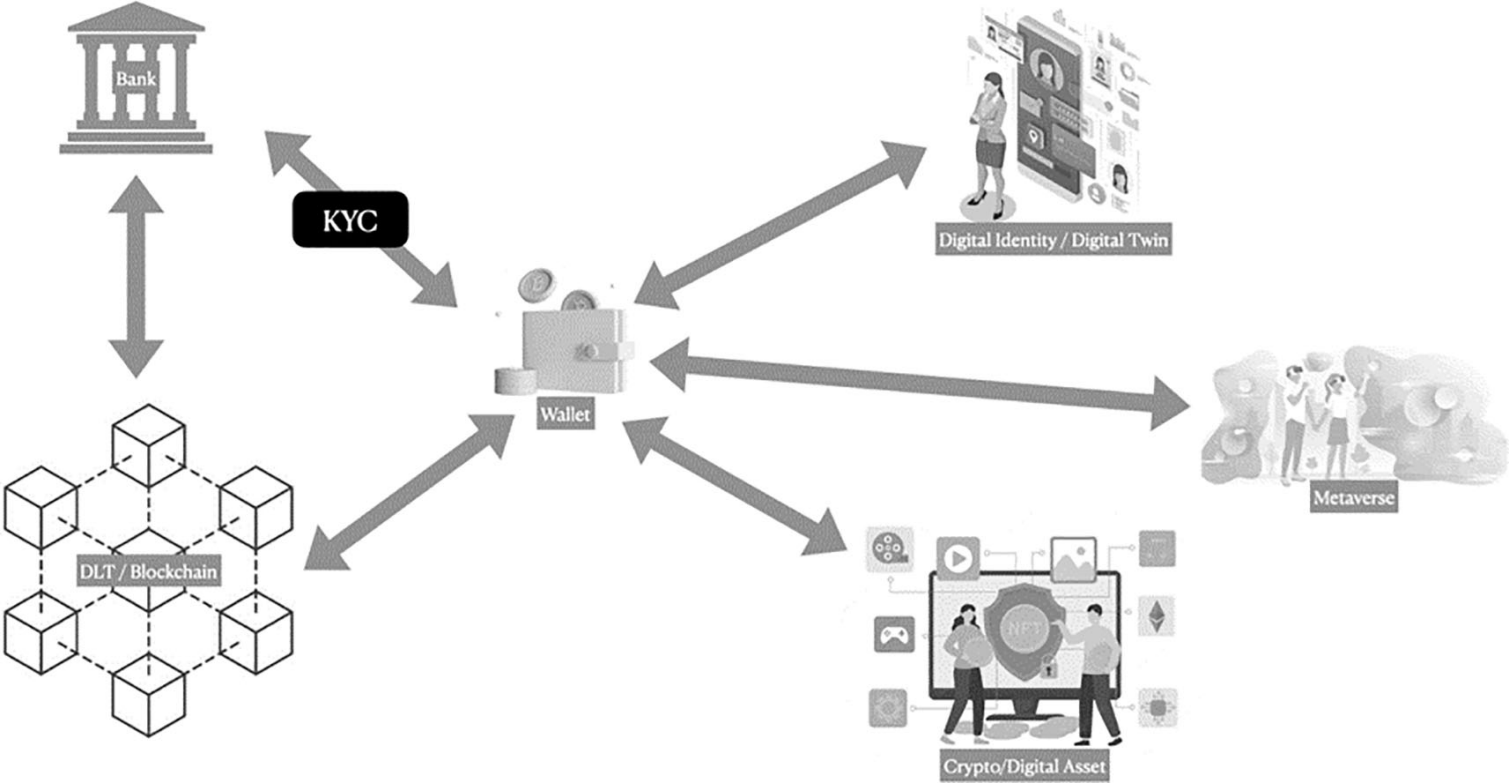
CBDC workflow illustration.

The recent surge in DeFi projects and ideas has prompted regulators, financial institutions, and banks to reassess their practices. The COVID-19 pandemic accelerated the digital economy, increasing online interactions and transactions. Digital natives or younger generations often seek opportunities for value creation among peers. Regulators, such as central banks, are adapting to the digital economy and peer-to-peer financial activities like DeFi by introducing Central Bank Digital Currencies (CBDCs). CBDCs enable effective monetary policy implementation, protect the public from malicious financial activities, and support innovation. ^46^ As mentioned above, it is hoped that CBDC could increase financial inclusions, enable faster and cheaper transactions, improve transparency, and reduce fraud. Notably, the most important regulator could have control over their monetary policy from the threat of the shadow economy. The CBDC workflow can be illustrated in Figure 2. ^47^

Figure 2 demonstrates how a commercial bank can facilitate the usage of CBDCs by verifying customer identities through a KYC mechanism. ^48^ Verified customers can then utilize a digital wallet as an interface to engage with DLT/Blockchain technology in the bank’s central or commercial back office. This wallet also enables customers to interact, transact, collaborate, create content, and play with their peers in a fully digital world, like the Metaverse. Additionally, the digital wallet provides secure and private digital identity protection against malicious internet activities and serves as a storage for crypto/digital assets, including NFTs, cryptocurrencies, and other assets.

**Figure 3.**
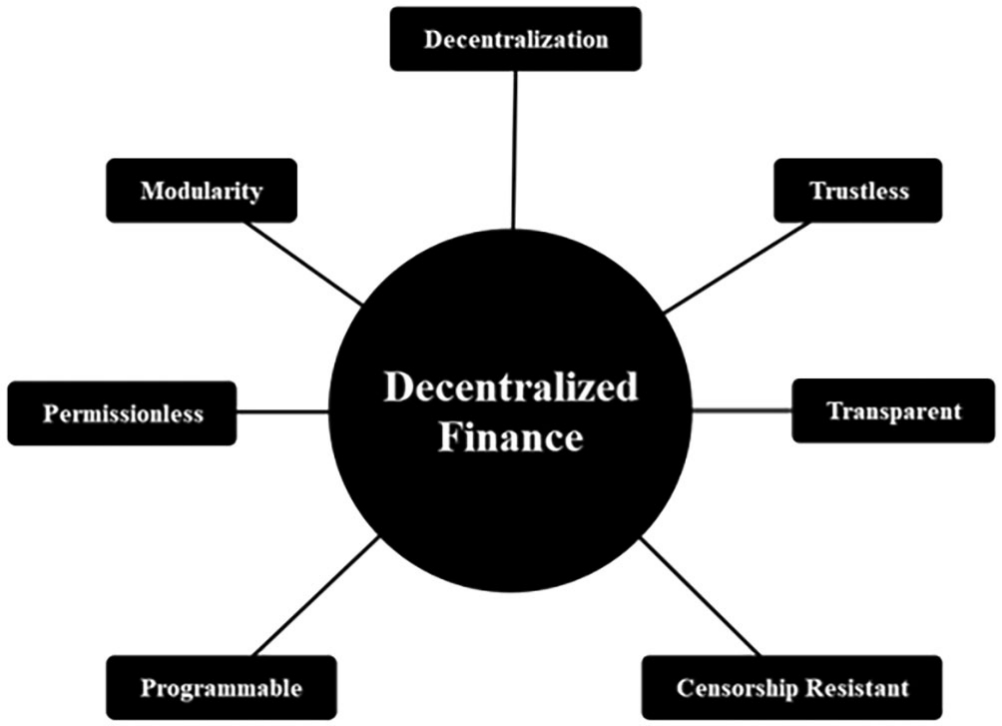
DeFi's characteristics.

We discuss several important DeFi products in the subsequent subsection. While DeFi characteristics are shown in Figure 3:

1. Decentralization: Refers to the capacity for conducting peer-to-peer transactions in DeFi, thus eliminating intermediaries’ roles.
2. Trustlessness: Smart Contracts remove the need for middlemen, enabling the automatic execution of agreement terms between users without manual verification of fulfillment.
3. Transparency: DeFi’s DLT system permits authorized users to monitor transaction activities, promoting data traceability, consistency, and immutability.
4. Censorship Resistance: Users can create unaltered transactions without interference from intermediaries.
5. Programmability: Pertains to the adaptability of Smart Contract users in setting agreement terms, limiting intermediaries to platform provider roles without the authority to dictate terms.
6. Permissionlessness: DeFi enables all users to conduct financial activities within the platform, without restrictions on participation.
7. Modularity: Refers to the ability of a contract to integrate with other contracts, such as combining savings and insurance, offering users flexibility in merging contracts based on preferences and needs.

**3.3.1.1 Decentralized exchanges**

A decentralized exchange (DEX) provides users with a platform to exchange assets without the involvement of third-party intermediaries, ^49^ unlike centralized exchanges (CEX), in which financial institutions mainly operate. One of the primary benefits of DEX is liquidity, ^50^ which refers to the ability to convert assets without a significant impact on the market price. The Automated Market Maker (AMM) mechanism determines DEX liquidity. DEX provides a way of transparency, symmetry information between parties, affordable to acquire financing, and proper risk management. Cryptocurrencies are currently the most commonly traded assets on DEX, but it is possible to trade fiat currencies, stocks, and other commodities in the future. For example, Uniswap, one of the most popular DEX platforms, allows users to swap between Bitcoin and Ethereum based on the Bitcoin to Ethereum ratio stored in the Smart Contract. ^51^ Users have complete control over their private keys in DEX, while financial institutions hold the keys in CEX. However, DEX can be challenging to comprehend since it is an emerging solution that lacks intermediaries. This makes it challenging to explain to new users.

**3.3.1.2 P2P lending**

P2P (Peer-to-peer) lending is a business model that facilitates borrowing and lending money between individuals through profit-driven online platforms, bypassing traditional financial institutions as intermediaries. P2P lending implementations cater to two user types: general platforms for any individual or small business and professional platforms for specific application domains. Blockchain technology can transform P2P lending in the following ways:

1. A transaction request is broadcasted to P2P nodes.
2. Nodes verify transactions and the user’s status.
3. Verified transactions are combined into a new data block for the ledger.
4. The new block is added to the existing blockchain.

Several prominent decentralized P2P lending platforms have emerged, such as:

1. *Aave*: A decentralized platform enabling lending and borrowing of various cryptocurrencies without intermediaries, utilizing smart contracts for P2P lending.
2. *Compound*: A decentralized platform for lending and borrowing various cryptocurrencies, leveraging smart contracts to execute loans in a trustless and secure manner.
3. *MakerDAO*: A decentralized platform allowing users to borrow a stablecoin called DAI using other cryptocurrencies as collateral, employing an intricate smart contract system to maintain DAI token stability.
4. *BlockFi*: Initially a centralized lending platform, BlockFi has expanded into decentralized lending with its BlockFi Interest Account (BIA), enabling users to earn interest on their cryptocurrencies by lending them to others.

**3.3.1.3 Equity crowdfunding**

Equity crowdfunding is a business model specifically designed to secure external funding for new ventures. ^52^ Unlike other forms of crowdfunding, equity crowdfunding primarily aims to connect entrepreneurs with investors who have an interest in investing in a particular firm(s). ^53^ Blockchain technology offers two significant advantages to equity crowdfunding ^54^:

1. Secure transaction of company ownership: Blockchain’s consensus algorithm and public key cryptography ensure that the transfer of company ownership is secure and tamper-proof. This adds an additional layer of trust and security for both entrepreneurs and investors.
2. Task automation: Blockchain technology allows for the automation of various tasks, such as interest payments and dividend distribution, through smart contracts. This automation eliminates the need for intermediaries, streamlining the process and reducing potential inefficiencies or transaction delays. This benefits both entrepreneurs and investors by simplifying the overall investment process and reducing associated costs.

*Republic, StartEngine,* and *Wefunder* are decentralized equity crowdfunding platforms enabling startups and small businesses to raise funds from diverse investors. *Republic* allows investments as low as $10 for company equity, *StartEngine* offers investment opportunities across industries, and *Wefunder* has helped over 600 companies raise over $200 million, empowering anyone to invest in startups they support.

**3.3.1.4 Cross-border payment**

Cross-border financial institutions enable payments between countries with different currencies. However, economic fluctuations resulting from events such as the 2001-2002 Argentine financial crisis, the 2008 global financial crisis, the 2014-2016 Russian financial crisis, the 2018-2020 US-China trade war, and the Covid-19 pandemic have disrupted cross-border payments. ^55^ The Society of Worldwide Interbank Financial Telecommunications (SWIFT) is a prominent organization in this space but its centralized nature results in prolonged transaction times and unclear operational fees. ^55^

*Ripple*, a blockchain-based project, addresses these issues through a decentralized peer-to-peer network for faster transactions and reduced fees compared to SWIFT. ^56^ *Ripple* also uses its own consensus algorithm called Unique Node List (UNL), where node operators select a list of “unique” nodes that they approve of. Nodes within the UNL are considered valid only if they have approval from other “unique” nodes, protecting the network from external attacks and preventing a majority vote from compromising nodes within the UNL. ^57^

**3.3.1.5 Stablecoin**

Stablecoins are a type of digital currency designed to maintain a stable value by pegging it to another asset, such as gold or fiat money. ^58^ Unlike other cryptocurrencies, which can exhibit significant price volatility, stablecoins provide a more predictable and reliable means of exchanging and storing value. Their development reflects market demand for a stable digital currency that emulates fiat money, which is typically controlled by regulators or central authorities.

While cryptocurrencies like Bitcoin exemplify blockchain technology, their high volatility and susceptibility to regulatory and informational influences have raised investor concerns. ^59^ In contrast, stablecoins exhibit low volatility and are increasingly used as an alternative to fiat currency in digital markets. ^58^ There are three primary categories of stablecoins based on their stabilization methods ^60^:

1. Asset-backed: Stablecoins pegged to and backed by assets held by private banks. Tether, for example, is an asset-backed stablecoin with its value pegged to the USD fiat.
2. Crypto-collateralized: Stablecoins whose value is pegged to other cryptocurrencies. The value of the collateralized cryptocurrencies exceeds the issued crypto-collateralized ones. *DAI* is an example, as its value is backed by USD and other cryptocurrencies like ETH (Ethereum’s cryptocurrency).
3. Algorithmic: This category of stablecoins does not rely on another asset for support. Instead, it uses oracle price feeds, algorithmic stabilization, and user participation (trading) to maintain its peg. *LUNA*, developed by Terra, a community-based blockchain platform, is an example of an algorithmic stablecoin. However, algorithmic stablecoins are often considered failures due to their remaining volatility, lack of widespread adoption, and vulnerability to unexpected events that disrupt supply and demand.

**3.3.1.6 Asset management**

The asset management firm’s objective is to record and transfer ownership of an asset based on a contractual agreement. ^61^ By establishing a decentralized system, blockchain facilitates asset transactions and maintains asset registries. This decentralized asset management system enables faster asset settlement processes, as there is no need for central authority validation, and it provides greater asset transparency as monitoring is performed through the Smart Contract. Asset management manages two kinds of assets ^62^:

1. Fungible. An asset can be exchanged with any other object, such as currency. For example, ten one-dollar bills are equal in value to a ten-dollar bill.
2. Non-fungible. It refers to a singular asset that possesses unique characteristics despite being identical to others. For instance, diamonds are classified according to their clarity, cut, color, carat, and certification. The diamond may differ from one to another, despite being referred to as “diamonds”.

Asset management firms can use permissioned or permissionless blockchains or both to improve their business operations. ^63^ Government officials can maintain the permissioned blockchain to verify the asset’s registration to prevent double-spending and ensure the legality of traded assets. In contrast, the permissionless blockchain represents the marketplace of traded assets. Asset management firms should consider using a permissioned blockchain to prioritize scalability and security. Meanwhile, if they plan to emphasize decentralization and accessibility, they may choose to use a permissionless blockchain.

There are three examples of decentralized asset management platforms in this paper. The first is *Melon Protocol*, which is a decentralized asset management platform that allows anyone to create, manage, and invest in digital asset portfolios. The platform uses smart contracts to ensure that assets are managed securely and transparently. The second example is called *Balancer.* *Balancer* is a decentralized asset management platform that allows users to create custom portfolios of cryptocurrencies. The platform uses an automated market maker algorithm to ensure that the portfolio’s value remains balanced. The last example is *Set Protocol.* It is a decentralized asset management platform that allows anyone to create, manage, and invest in tokenized portfolios of cryptocurrencies. The platform uses smart contracts to automate portfolio management tasks and ensure the portfolio complies with them.

**3.3.1.7 Insurance**

Fraud has been a significant concern in the conventional insurance sector. The FBI estimates losses in the United States alone to exceed $40 billion (about $120 per person in the US), or about $120 per person. This issue, combined with several drawbacks in the insurance industry, such as lack of customization, bureaucratic complications, limited transparency, and customer dissatisfaction, calls for innovation. Blockchain technology has the potential to enhance the efficiency of the insurance industry in numerous ways.

1. Fraud elimination: Companies like *Everledger* utilize the blockchain to create a global registry for items like gemstones, making fraudulent transactions more difficult.
2. Automated claim management process: Integrating claim management data enhances visibility for insurers, brokers, and reinsurers.
3. Reinsurance: Smart Contracts facilitate clear and open systems between reinsurers and insurers.
4. Transparency: Blockchain improves claim tracking, product verification, and service authentication transparency.
5. Enhanced consumer management: Blockchain enables personalized insurance products catering to specific customer needs.
6. Data analysis: Insurance companies can make better business decisions by analyzing customer data within the blockchain network, such as credit information, policy details, and accident environmental data.

Three examples of blockchain-based insurance platforms include *Nexus Mutual*, a decentralized insurance firm offering coverage for smart contract failures on the Ethereum network; *Etherisc*, a decentralized insurance platform allowing individuals and businesses to create and purchase insurance products using smart contracts, covering various needs like flight delay and crop insurance; and *InsurAce*, a decentralized insurance platform providing coverage for a range of DeFi products and services, using a mix of on-chain and off-chain data for policy pricing and claim assessment.

### 3.4 Threats

**Figure 4.**
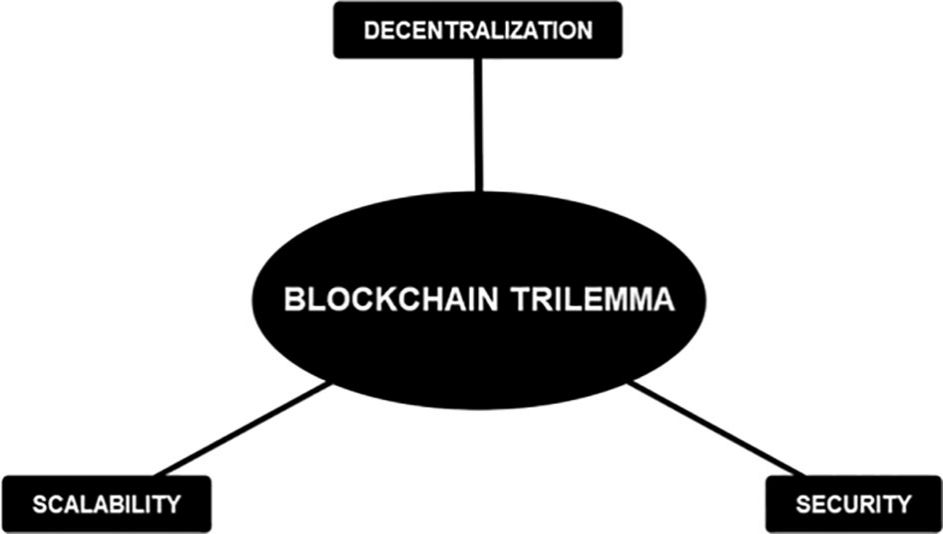
Blockchain Trilemma.

Notwithstanding the substantial advancements in the financial sector, blockchain technology remains encumbered by certain limitations that warrant attention. Inadequate management of these limitations may result in unfavorable repercussions for organizations that adopt this technology. It is crucial to examine the blockchain trilemma when discussing this subject matter. Figure 4 presents a graphical depiction of the blockchain trilemma for elucidation purposes:

The trilemma theorizes that a blockchain implementation is challenging to cover all three attributes. ^64^ The attributes consist of decentralization, security, and scalability. Decentralization refers to the distribution of the network among numerous nodes and is not centralized. Security means that the network is resistant to tampering and attack. The scalability attribute refers to the situation where the network can handle lots of transactions in a short period of time. Currently, a blockchain network is only able to cover two of the attributes. It challenges the developers to establish a balance between those three attributes to the specific needs of a product/service. We have identified five threats associated with implementing blockchain in the financial industry. The threats are listed as follows.

**3.4.1 Scalability and speed**

Scalability and speed are two interrelated threats in blockchain utilization. Scalability refers to the blockchain’s ability to handle the increased amount of workload. Speed is the amount of time a system requires to conduct a certain task. Those threats are related since a system is addressed as scalable if it can handle a workload increase within a specific amount of time. In several cases, blockchain networks may be unable to handle the increased workload, thus slowing the whole network. As more transactions are added to the blockchain network, the slower it gets. Fortunately, researchers and platform developers are developing solutions to address these threats. It aims to make blockchain more practical and efficient to face the increased workload.

**3.4.2 Security**

Several factors contribute to the risk of security threats in the digital realm. One such factor is the susceptibility of consensus algorithms, like Proof of Work (PoW), to manipulation. Attackers can gain control over the system by amassing 51% of its computational power, which enables them to dictate their own rules. However, this level of control is challenging to achieve due to the vast number of network participants. Phishing is another security threat, where scammers deceive victims into investing in counterfeit cryptocurrency wallets. Once victims share their information with the fraudulent wallet, they cannot access their funds without the private key. Lastly, a Sybil attack poses a significant security threat by creating numerous false identities to seize network control. If successful, the attacker can exploit the network with impunity.

**3.4.3 Cost**

Although blockchain can potentially reduce transaction costs, users in FinTech must pay service fees to miners rather than a financial institution, with variable costs depending on the effort required and traffic. Fees incentivize miners to include transactions in the next block, with the amount dependent on the demand and supply of block space. High demand leads to increased fees, which contribute to rising costs. Hardware prices, bandwidth requirements, and network synchronization complexities are also cost factors. These factors have hindered widespread adoption, however newer innovations like Layer 2 (L2) mechanisms offer a faster and more affordable implementation.

**3.4.4 Regulation**

Insufficient regulations can negatively impact the establishment of new products, leading to disorder and increased criminal activities. Therefore, it is important to strike a balance between regulation and innovation. It is also important to note that the level of regulation may differ among countries, and weak regulations and limited user knowledge regarding blockchain can make it easier for criminals to carry out illegal activities. Regulators must play a vital role in establishing regulations regarding blockchain-based digital assets to prevent negative consequences. If authorities fail to regulate blockchain, it could lead to various negative outcomes. Firstly, it could facilitate money-laundering activities due to the anonymity provided by blockchain transactions, allowing criminals to obscure their funds’ origin. Secondly, customers may be vulnerable to blockchain scams due to the technology’s complexity and lack of protection. Therefore, regulators should establish appropriate rules to safeguard citizens. Lastly, the lack of standardization and regulation could result in interoperability issues between different blockchain networks, hindering or even preventing transactions between companies. The regulations of blockchain technologies vary among developed and developing countries. Developed countries tend to have a clearer legal framework for blockchain and a higher level of blockchain adoption than developing countries. Also, developed countries tend to have clearer tax regulations, whereas developing countries may still determine the tax procedure.

**3.4.5 Environment and energy**

The energy consumption of blockchain technology, specifically the PoW mechanism, has raised concerns about its negative environmental impact. In fact, a single Bitcoin transaction can consume as much as 1,499 kWh of electricity due to the intense competition between miners to solve computational problems and earn rewards. ^65^ This increased demand for electricity has led to more fossil fuel consumption, contributing to increased carbon emissions and pollution. The widespread use of PoW as a consensus mechanism in cryptocurrencies has further compounded these environmental issues, necessitating alternative consensus algorithms promoting environmental sustainability. Additionally, the issue of hardware waste has significantly affected environmental sustainability, as blockchain companies often require the continuous procurement of new hardware with a limited lifespan. Furthermore, the high energy requirements for mining activities have led to a concentration of mining activities in regions with low energy costs or favorable tax policies, leading to centralization, contrary to the decentralized philosophy underpinning blockchain technology. Therefore, it is crucial to address these environmental concerns to ensure blockchain technology’s sustainable growth and development.

## 4. Threat solutions

Numerous researchers have proposed potential solutions to mitigate the threats described in the preceding section ( subsection 3.4). It is worth noting, however, that these solutions are not the only viable means of addressing these threats. Financial institutions can implement the suggested solutions or explore superior alternatives that may emerge in the future. Therefore, it is critical for financial institutions to remain vigilant and flexible in their approach to security, ensuring they effectively tackle the constantly evolving nature of these threats. Financial institutions must continuously assess their security measures and invest in innovative approaches that provide the best possible protection against cybersecurity threats. This proactive approach will ensure they remain ahead of the curve in the ongoing battle against cybercriminals. By prioritizing cybersecurity, financial institutions can better safeguard their customers’ sensitive information and maintain their reputation for trust and reliability.

### 4.1 Scalability and speed

Researchers have proposed solutions to address scalability and speed issues in blockchain systems, particularly Layer 1 (L1) platforms like *Bitcoin* and *Ethereum*, which cannot be easily modified. A solution called Layer 2 (L2) has been developed to overcome these limitations, which involves aggregating transactions before writing them to L1. Several techniques have been developed for L2, including Simplified Payment Verification (SPV), which reduces data usage compared to traditional methods, Lightning Network (LN), which uses micropayment channels to update balances continuously, ^66^ and sharding, a technique that partitions data among multiple nodes. ^67^ Sharding divides the network into smaller shards, each responsible for processing a subset of transactions, reducing the burden on individual nodes and increasing transaction processing speed. Additionally, sharding offers benefits such as sublinear communication, higher resiliency, rapid committee consensus, secure configuration, fast cross-shard verification, and decentralized bootstrapping. Notably, *Rapidchain* and *Ethereum 2.0* are among the leading platforms that have adopted sharding.

### 4.2 Security

To mitigate the risk of a 51% attack, various consensus algorithms such as PoS, Unique Node List (UNL), Proof of History (PoH), and Proof of Authority (PoA) can be used instead of the traditional PoW. PoS, for example, reduces the possibility of a malicious entity taking over the network by making it almost impossible to perform a 51% attack. *Peercoin* is an example of a blockchain product that utilizes the PoS mechanism. ^68^ UNL is another solution that protects the network from external attacks by preventing malicious entities from entering the network without being recognized as the node operator. PoH is a consensus algorithm proposed by *Solana*, where every transaction has a unique hash and count, serving as a real-time record that miners can use to reconstruct transactions based on their historical records, increasing resilience against possible attacks. ^69^ PoA is another type of consensus where miners stake their reputation, rather than coins, to be recognized as a validator. ^70^ This means they are not anonymously operated, unlike in PoW and PoS, making it easier to detect conspiring parties and increasing resistance to 51% of attacks.

However, security threats may also arise from the lack of user awareness, rather than the system. Therefore, there are several solutions to prevent attacks. The first is to avoid suspicious financial investments and invest in registered financial institutions, which are less likely to conduct scams. Raising awareness of various scamming methods, such as phishing, pop-ups, and spoofing, is another solution to increase user awareness and prevent attacks. ^23^

**Figure 5.**
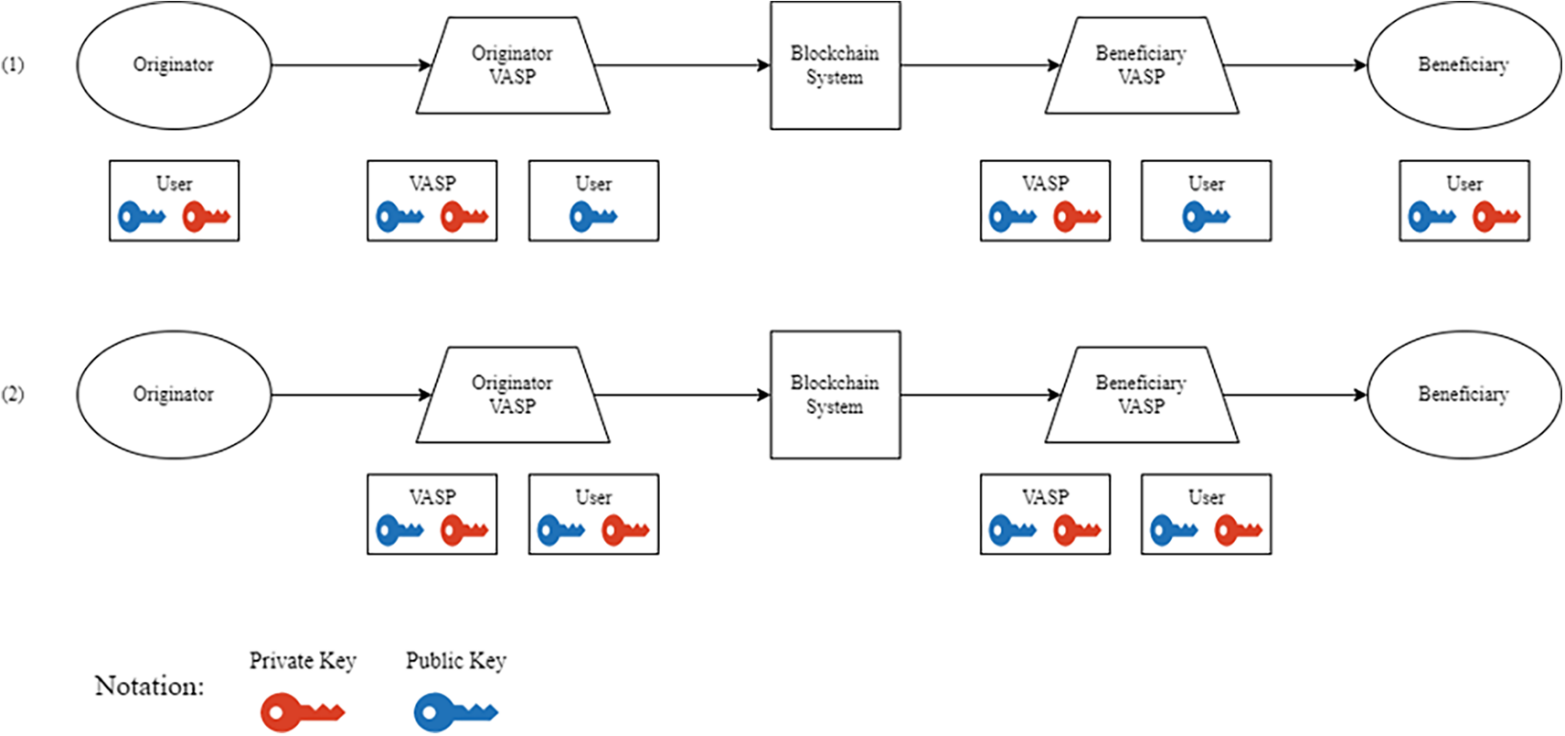
VASP Public-Private Key Management Scheme.

Over the course of several years, the Financial Action Task Force (FTAF) has implemented various changes to its policies, ultimately resulting in the release of Recommendation No. 15. This recommendation mandates that cryptocurrencies and their affiliated financial institutions disclose information about the entities and recipients involved in digital asset transactions, which is commonly referred to as the travel rule. ^71^ However, Virtual Asset Service Providers (VASPs) - as financial institutions - have encountered obstacles when attempting to apply the travel rule to blockchain-based digital assets. The core issue is that VASPs struggle to manage the cryptographic keys associated with their customers, as digital assets on a blockchain are directly controlled through a private-public key mechanism. To address these challenges, Figure 5 outlines two potential approaches ^68^:

1. Non-custodian: This approach utilizes a secure method where the parties involved in the asset transfer - the originators and beneficiaries - hold both private and public keys. At the same time, the VASP only has access to their public keys. This ensures that the VASP’s involvement is limited to monitoring the transaction and does not involve intermediation. By complying with the travel rule, this approach empowers financial institutions to validate and verify transactions before they are executed, thus enhancing the security and soundness of the financial system.
2. Custodian: This refers to a custody-based approach, where the VASP assumes responsibility for the secure storage of both the public and private keys, and neither the originator nor the beneficiary has direct ownership of the keys. This method enables financial institutions to manage the transfer of their clients’ assets and is commonly used by blockchain-based exchanges such as *Binance, Coinbase,* and *Uphold.* By employing a robust custody mechanism, financial institutions can ensure their clients’ assets are secure and safeguarded against potential threats or loss.

### 4.3 Cost

Ethereum 2.0 provides a viable solution to address the high costs associated with blockchain technology. Through the use of PoS and sharding, Ethereum 2.0 reduces the computational requirements of blockchain transactions, thereby lowering transaction costs. ^69^ Integrating the Beacon Chain, a new type of consensus layer, with the existing PoW layer in Ethereum 2.0 represents a unique approach that eliminates the need for traditional PoW consensus, resulting in faster processing times and significantly reduced transaction costs. ^70^ Miners can optimize gas usage to reduce costs by improving code efficiency and consolidating multiple transactions into a single batch. However, addressing the cost-related challenges in the blockchain industry requires a multifaceted approach that involves technological solutions and market reforms. As blockchain innovation continues to evolve, it presents opportunities for new platforms to emerge with lower costs than existing ones, making the industry more accessible and widely adopted. The birth of various new blockchain platforms is motivated by the need for cost reduction, most of which are L2 solutions, as we mentioned in subsection 4.1.

### 4.4 Regulation

Regulating blockchain technology is a multifaceted issue that presents challenges due to the diverse regulatory landscape across jurisdictions. Nonetheless, several potential solutions could be implemented globally to address this challenge. The first solution involves governments prioritizing the development of blockchain regulations that promote data privacy, anti-money laundering, and consumer protection. Through such regulations, governments can ensure blockchain operations align with legal frameworks and safeguard stakeholder interests. Secondly, creating regulatory sandboxes offers a controlled environment for blockchain companies to experiment with regulatory approaches in collaboration with regulators. This approach enables a balance between innovation and regulation. ^72^ Thirdly, international cooperation among countries is essential, given the global nature of blockchain technology. Governments can collaborate to establish global standards and coordinate regulatory efforts, promoting innovation, accountability, and consumer protection. Finally, adopting CBDC represents a potential solution for regulating blockchain technology in the financial industry. By issuing a regulated form of cryptocurrency, central banks can monitor and enforce compliance with regulations while enhancing transaction transparency and preventing threats such as money laundering. However, it is important to balance innovation and regulation to mitigate potential risks and promote the industry’s growth.

### 4.5 Environment and energy

Several methods can be utilized to address environmental and energy issues associated with blockchain technology. The first method is the implementation of the PoS algorithm, which lowers the mining difficulty for each node and increases the speed of the mining process based on the number and duration of coins held by the node, ^7^ resulting in fewer participating nodes than PoW. The second method involves the use of permissioned blockchains, which limit the number of parties in the network, ultimately reducing energy consumption. Transitioning mining operations to renewable energy sources such as solar, water, and wind can also be a potential solution to reduce the carbon footprint of the mining process. In addition, blockchain-as-a-service (BaaS) can be leveraged as a means of reducing energy consumption, as it allows companies to outsource their blockchain networks to third-party providers, thereby reducing the need for companies to establish and maintain their own energy-intensive blockchain networks. Finally, raising awareness and education about the impact of blockchain on the environment and energy sustainability is essential. Individuals and organizations can take steps to reduce their environmental impact and promote energy sustainability through their use of blockchain technology. By adopting a multifaceted approach that integrates technological and social solutions, we can mitigate the negative impact of blockchain on the environment and energy sustainability.

## 5. Discussion

Blockchain technology has gained significant attention recently, particularly in the financial industry. This is due to its potential benefits, such as low-cost financial services, high security and privacy, efficient financial transactions, high return on investment, and transparency. Two key drivers of blockchain technology are Smart Contracts and DLT, which are essential for establishing a decentralized system. The decentralized nature of blockchain technology has the potential to promote financial inclusion by providing low-cost financial services to the unbanked and underbanked populations. This has the potential to revolutionize how financial services are offered, particularly in developing countries where traditional financial services may be costly and inaccessible.

Furthermore, blockchain technology can offer high security and privacy, protecting users’ sensitive financial information. Efficient financial transactions, such as remittances, can also be facilitated using blockchain technology. Blockchain-based solutions can offer faster transaction processing times and lower transaction fees than traditional financial institutions. Additionally, the programmable nature of blockchain technology allows for the creation of programmable assets and currencies, which can support new financial instruments and business models.

Despite its potential benefits, the adoption of blockchain technology in the financial industry faces several challenges. One of the significant issues is the complexity of the blockchain system, which requires significant technical expertise to implement and maintain. For example, how to transform the business process into the blockchainable one. ^73^ The lack of standardization and regulation also presents a challenge, as it can hinder the technology’s adoption in the industry. Other prominent issues that we already mentioned and have been discussed extensively in the section threat and its solution, including cost, speed, and scalability issues.

To overcome these challenges, there is a need for education and awareness programs to provide human resources with the necessary knowledge and skills to implement and maintain blockchain technology. Standardization and regulation efforts can ensure that blockchain technology is governed within a legal framework protecting all parties involved. It is important to note that the problems and solutions discussed in this context cannot be universally applied. Each country may have its own rules and regulations for governing blockchain technology within its jurisdiction. However, as blockchain technology continues to grow and expand beyond borders, there may be a need for regulation that spans multiple countries. It is crucial that regulators must find a balance that ensures the safety and security of all parties involved to prevent any negative outcomes from arising.

## 6. Conclusion

We have carefully crafted a taxonomy based on four dimensions, which we believe are essential in understanding and mapping the blockchain-based financial industry. We have provided detailed explanations of each dimension, including the key drivers, benefits, products, and threats associated with blockchain technology. We have also analyzed the interdependence between each dimension to understand better how they can impact one another. It is important to note that our taxonomy’s dimensions may not be exhaustive, as the blockchain-based financial industry is constantly evolving. However, our paper provides a comprehensive overview of the conditions at the time of writing. We have made a conscious effort to highlight the most significant aspects of blockchain technology in finance.

One of the key advantages of blockchain is its potential to increase business process efficiency and drive innovation in the financial industry. However, establishing adaptive regulations is crucial to ensure that users and financial institutions utilize blockchain technology securely and responsibly. It is possible that our paper may not have captured all dimensions of the blockchain-based financial industry, given the rapidly evolving nature of this technology. Therefore, future research could delve deeper into exploring the gaps to gain a more profound understanding of blockchain’s role in FinTech companies. Overall, our paper aims to provide valuable insights into the blockchain-based financial industry and its potential for disrupting traditional financial systems.

## Author contributions

Conceptualization: A.A., Data Curation: S.S. and A.A., Formal Analysis: A.A. and S.S., Methodology: A.A., Resources: S.S., Supervision: A.A., Validation: A.A. and S.S., Visualization: S.S., Writing – Original Draft Preparation: S.S., Writing – Review & Editing: A.A. and S.S.

## Data Availability

No data are associated with this article.
