# Supplementary material for: The Taxonomy of Blockchain-based Technology in the Financial Industry
Source: F1000Res. 2023 Jul 18;12:457. Originally published 2023 May 2. [Version 2] doi: 10.12688/f1000research.133518.2 (PMC10394392; doi:10.12688/f1000research.133518.2)
Supplement: Peer review history

# Peer review: Reviewer response for version 2

**DOI:** 10.5256/f1000research.153064.r188297

**Published:** 2023-08-01

**Authors:** Wisnu Uriawan

**Affiliations:** 1 Universitas Islam Negeri Sunan Gunung Djati Bandung, Bandung, West Java, Indonesia

This paper has already been revised comprehensively according to my suggestions.

Is the review written in accessible language?

Yes

Are all factual statements correct and adequately supported by citations?

Partly

Are the conclusions drawn appropriate in the context of the current research literature?

Partly

Is the topic of the review discussed comprehensively in the context of the current literature?

Yes

Reviewer Expertise:

NA

I confirm that I have read this submission and believe that I have an appropriate level of expertise to confirm that it is of an acceptable scientific standard.

# Peer review: Reviewer response for version 2

**DOI:** 10.5256/f1000research.153064.r188296

**Published:** 2023-07-26

**Authors:** Giulio Caldarelli

**Affiliations:** 1 Department of Management, Universita degli Studi di Torino, Turin, Piedmont, Italy

I read the revised article. It appears that the authors improved the paper according to my and other reviewer comments. I suggest to approve the paper as it is. I do not have further comments.

Is the review written in accessible language?

Yes

Are all factual statements correct and adequately supported by citations?

No

Are the conclusions drawn appropriate in the context of the current research literature?

No

Is the topic of the review discussed comprehensively in the context of the current literature?

Partly

Reviewer Expertise:

Blockchain, Oracles, and Decentralized Finance.

I confirm that I have read this submission and believe that I have an appropriate level of expertise to confirm that it is of an acceptable scientific standard.

# Peer review: Reviewer response for version 1

**DOI:** 10.5256/f1000research.146513.r171682

**Published:** 2023-05-22

**Authors:** Wisnu Uriawan

**Affiliations:** 1 Universitas Islam Negeri Sunan Gunung Djati Bandung, Bandung, West Java, Indonesia

The paper describes The Taxonomy of Blockchain-based Technology in the Financial Industry. The taxonomy of this context is interesting, and it is worth to be investigated.

 Still, the paper seems more like a presentation of a theory report than a research paper. In the present form, the manuscript appears more like a white paper.

 In particular, I have the following significant comments:

- The advantages and disadvantages of adopting blockchain technology must be better described than other types of blockchain or other solutions not using the blockchain. There is a concise analysis for each section, but more is needed.
- I recommend the authors create a specific section to analyse the advantages and disadvantages of adopting blockchain technology in this context.
- The potential risk and vulnerabilities of the proposed approach should be discussed more. The principal vulnerabilities of the proposed system should be described together with the related countermeasures.
- A new section could be created, or vulnerabilities and countermeasures could be discussed.
- It would be interesting to link the DeFi concepts in the marketplace with the design description and how the taxonomy could be enhanced using blockchain technology and its records history.
- It would be helpful to provide more details in Section 4 on the potential standards and protocols that could be used to implement the proposed approach.

Is the review written in accessible language?

Yes

Are all factual statements correct and adequately supported by citations?

Partly

Are the conclusions drawn appropriate in the context of the current research literature?

Partly

Is the topic of the review discussed comprehensively in the context of the current literature?

Yes

Reviewer Expertise:

The blockchain technology

I confirm that I have read this submission and believe that I have an appropriate level of expertise to confirm that it is of an acceptable scientific standard, however I have significant reservations, as outlined above.

# Response

**Published:** 2023-07-14

**Authors:** Andry Alamsyah

**Affiliations:** Telkom University, Indonesia

1.    The advantages and disadvantages of adopting blockchain technology must be better described than other types of blockchain or other solutions not using the blockchain. There is a concise analysis for each section, but more is needed.

*Thank you for the suggestion. After we describe the advantages and disadvantages of financial industry blockchain implementation in subsection 2.2, we add a concise summarization in Table 4. However, it is worth noting that we only focus on the key features related to the financial industry; thus, it might not include all aspects.*

 2.    I recommend the authors create a specific section to analyse the advantages and disadvantages of adopting blockchain technology in this context.

*Thank you for the advice. Since our primary objective is focusing on the business processes and products/services in the financial industry, adding a specific section for blockchain technology will devise the focus more on the technological side. Moreover, adding the requested explanation in Table 4 is already sufficient to support the paper's objective. We will consider this recommendation in the future taxonomy development focusing on technological aspects, which we write in the future research suggestion in the conclusion section.*

 3.    The potential risk and vulnerabilities of the proposed approach should be discussed more. The principal vulnerabilities of the proposed system should be described together with the related countermeasures.

*We have dedicated section 4 specifically to discuss potential risks/vulnerabilities of blockchain technology usage. Each subsection discusses the state of the idea of how to resolve the problem and how to mitigate the risks in practice.*

 4.    A new section could be created, or vulnerabilities and countermeasures could be discussed.

*The threat solutions section is intended to complement the main idea of taxonomy creation. It is not the main idea of the paper. Using Table 4 and the discussion on threats solutions section is enough for the complementary discussion. Nevertheless, we thank the reviewer for pointing out this idea; we will consider writing a new taxonomy paper specifically for the technological aspects.*

 5.    It would be interesting to link the DeFi concepts in the marketplace with the design description and how the taxonomy could be enhanced using blockchain technology and its records history.

*The purpose of the study is to construct the taxonomy for a higher general purpose (audience) which has no or little prior knowledge about blockchain technology and how this technology can achieve several abstractions that were impossible to be implemented before, such as peer-to-peer transactions, transparency, and automation (smart contract). This new perspective will inspire creativity that sparks many crypto industry marketplaces today where most of them in the form of DeFi and GameFi. For the discussion to add future taxonomy enhancement, we have added the following suggestion in the conclusion section*

*"Therefore, future research could delve deeper into exploring the gaps to gain a more profound understanding of blockchain's role in FinTech companies. Another approach is to develop the taxonomy based on blockchain technical aspect exploration."*

 6.    It would be helpful to provide more details in Section 4 on the potential standards and protocols that could be used to implement the proposed approach.

*Thank you for the suggestion, we have mentioned the trade-off of blockchain protocol choice based on the blockchain trilemma in section 4. The practical use case might differ between implementations, so we would prefer to underline the standard principle in resolving threats in the blockchain trilemma concept: speed/scalability, cost, and security.  We avoid mentioning the blockchain protocol choice since we can not keep up with the dynamic development of blockchain protocols. For example, today, the fastest blockchain protocol is Solana at the rate of 50000 transactions per second, but it might soon change with the emergence of new blockchain protocols.*

# Peer review: Reviewer response for version 1

**DOI:** 10.5256/f1000research.146513.r171680

**Published:** 2023-05-22

**Authors:** Giulio Caldarelli

**Affiliations:** 1 Department of Management, Universita degli Studi di Torino, Turin, Piedmont, Italy

Dear Author,

 Thanks for submitting your manuscript to F1000Research. As you know, the journal is reputable and indexed in the main scholarly databases.

 The manuscript you submitted concerns a taxonomical analysis of Blockchain based applications in the financial sector. It is undoubtedly a useful resource for scholars and practitioners working in the field.

 Although I believe it is an interesting contribution, some changes are necessary, in my opinion, for the article to be understandable to a broader audience. Please accept some suggestions to improve the paper.

**Introduction**

1. In the introduction, some words are closed in quotation marks (“ “) and others are not. I’m referring to “internal, provider oriented and customer-oriented digitalization”. Please consider a consistent use of quotation marks for all of them.
2. “but first we need to explain what blockchain is”. Consider removing this sentence. It is more appropriate for a blog article than an academic paper. If you believe you need to explain blockchain, go ahead and explain it directly. By the way, I believe the ledger is made of blocks and is secured by cryptographic proofs.
3. The revolution brought by blockchain to the organizational structure can be heterogeneous and not only due to the DAOs. I believe you need to specify also the organizational structure of what type of company. Did you mean the financial ones?
4. “Blockchain is the technology behind Bitcoin”. I heard this sentence a lot, but I don’t think is very accurate. There are many technologies behind Bitcoin. Blockchain sometimes is used as a synonym for Bitcoin. We cannot say that blockchain is a technology. It is more a concept thanks to which we refer to bitcoin-like ecosystems. Consider removing the sentence
5. “Another blockchain product in the financial sector is Centralized Finance (CeFi)”. Its often used as synonymous with Traditional Finance (TradFi). I hardly hear CeFi as a goal of blockchain implementation. So please specify what you wish to intend here.
6. A critical part that is missing in the Introduction is the significance of the study. Do you believe that among all the published papers on DeFi, there is still a need for a review? I truly read many, many review papers on Decentralized Finance. Why do you believe your research is fundamental at this stage? What are you offering that other published papers have not? This is a critical point also to understand the motives behind the paper type you have selected. Is a classic review appropriate for the contribution you are proposing?

**Literature Review**

 The idea consists in a review of papers which is coherent with the scope of the paper, I believe. What I cannot understand is the practical method, thanks to which you took the articles. You said that you picked 73 articles from some publishers, but how? Why those publishers? What is the ratio? What is the academic approach? Where did you put the mentioned keywords? Have you queried some academic databases? You say that you gathered information from crypto projects. Why did you add data from them? Was the data retrieved insufficient? How did you find them? What are the criteria? What is the source? Why is the source selected? Is there any filtering? It is true that this article is intended to be a simple review paper, but what you are proposing here is probably more consistent with a systematic review. Being a paper that tries to clarify and harmonize the taxonomy, it probably requires a well-written and explained methodology. Do you know other taxonomical paper that use a simple review explained in that way?

 A paper that can be of inspiration for what you are writing here is the following one:

 Tasca P., Tessone C.J., “A Taxonomy of Blockchain Technologies: Principles of Identification and Classification “ https://ledger.pitt.edu/ojs/ledger/article/view/140

 Although they are not using a systematic review, they give a specific contextualization of their approach.

 Therefore, my suggestion is to remove the literature review paragraph and write a section that clearly describes how you organized your research on taxonomy. Explain thoroughly and carefully how you got the articles, sources, and those interviews you mentioned in the abstract. Again, since you also mentioned interviews, a simple review paper type feels inappropriate. Consider giving all possible details on this data. If possible, provide a link to a repository. Explain your research idea extensively, the gap you are covering, and how methodologically you are going to cover it. Build on previous research articles and be inspired by other reviews in the field. Although not required for simple review, it would be better if what you achieve is replicable.

**The Taxonomy**

 This section is interesting. However, it really needs to be extensively explained and well-linked to the methodology section. Otherwise, it is not even clear how you made the graphical word map.

**Discussion**

 The discussion appears to be just sketched. Given the length and heterogeneity of the information provided, readers probably expect some more elaboration from the authors.

 Unfortunately, without a thorough methodological explanation, I cannot continue the review process properly. Therefore, I must suggest rewriting the methodological part so that the rest of the content can be evaluated. Of course, it is vital to explain in advance the significance of the paper and the reason why at this stage of research and for this specific goal, a simple review paper is selected.

 Good luck with your research!

Is the review written in accessible language?

Yes

Are all factual statements correct and adequately supported by citations?

No

Are the conclusions drawn appropriate in the context of the current research literature?

No

Is the topic of the review discussed comprehensively in the context of the current literature?

Partly

Reviewer Expertise:

Blockchain, Oracles, and Decentralized Finance.

I confirm that I have read this submission and believe that I have an appropriate level of expertise to confirm that it is of an acceptable scientific standard, however I have significant reservations, as outlined above.

## References

[1] : A Taxonomy of Blockchain Technologies: Principles of Identification and Classification. *Ledger* .2019;4: 10.5195/ledger.2019.140 10.5195/ledger.2019.140

# Response

**Published:** 2023-07-14

**Authors:** Andry Alamsyah

**Affiliations:** Telkom University, Indonesia

**Introduction**

 1.    Thank you for pointing out the inconsistencies; we decide to remove the quotation marks (“ “) and modify the sentence accordingly.

 2.    Thank you for the suggestion; we have modified the statement to follow the formality of the academic paper. We also alter the misleading statement into 

 “… consists of a chain of block secured by cryptographic proof to secure its integrity.”

 3.    Thank you for the critical opinion on DAO usage. We have stated to limit only the organization structure; thus, mentioning the DAO conceptual approach is relevant here.  

 What we meant here is the usage DAO conceptual approach, which is decentralized and autonomous to generate/spark innovation. To make matters clear, we have modified the text in the 3rd paragraph as follows

 “…The revolution brought by blockchain to the organizational structure is through Decentralized Autonomous Organization (DAO) idea. The DAO concept offers a decentralized approach rather than a pyramid hierarchy, often used in conventional institutions, including the financial industry. The decentralization and autonomous concept also drive the innovation of numerous new products, eliminating intermediaries’ functions. For instance, a digital cryptocurrency operated on a decentralized peer-to-peer network, called bitcoin..” 

 4.    Thank you for the critics. We have removed the “blockchain is the technology behind the bitcoin” statement. 

 5.     Thank you for the correction; we have added TradFi to the CeFi term.

 6.    Thank you for the constructive critics. We have found several precedent articles regarding blockchain in the financial industry and DeFi taxonomy—notably a publication from Puschman and Huang-Sui. We have mentioned these publications in the 5th paragraph of the introduction section. We explained in detail the difference between our paper and the previous article in scope, control, financial instruments, interoperability, risk, and regulation. We underline that since blockchain in the financial industry is still nascent, the public would benefit from different approaches and diverse literature views. 

**Literature review**

 1. We have modified the previous section 2 about the literature review section into the methodology and literature review section. Subsequently, we divided this section into subsections 2.1 Methodology and 2.2 Literature Review.

 In the methodology, we clarify that we do not choose specific publishers to construct the taxonomy construction.  The idea is to collect information from scholars, industry, and regulators using related keywords such as blockchain technology, financial industry, decentralized finance, token economy, application, implementation, and blockchain for business processes. Several important questions are asked and explored to clarify the scope further to construct the taxonomy. The questions are shown in the 2nd paragraph of subsection 2.1. Keywords and questions become the fundamental idea of taxonomy construction. 

 We add Table 2 to describe the academic publication source and its ratio. Also, we categorize the academic publication into 3 categories, which we explain in 3rd paragraph of subsection 2.1. Thus, the publication category is shown in Table 3. 

 We also explain clearly the role of literature resources from the industry, including several DeFi projects and the regulator (CBDC and BIS). 

 2.  Thank you for the constructive suggestion. We have altered section 2 into the research methodology and literature review to cover your suggestion regarding how we achieve this research and provide the necessary coverage for the literature review. We also decided to remove the expert interview sections since the majority of our sources come from the literature. Initially, we planned to use domain expert input to strengthen our result. However, we found, it is sufficient from the available literature review.

**Taxonomy**

 Thank you for the valuable suggestion. We have added the methodological section, in line with research motivation, literature research criteria through keywords and essential questions, and publication categorization. We hope the explanation can justify the taxonomy construction.
